# Default Mode Network in the Effects of Δ9-Tetrahydrocannabinol (THC) on Human Executive Function

**DOI:** 10.1371/journal.pone.0070074

**Published:** 2013-07-31

**Authors:** Matthijs G. Bossong, J. Martijn Jansma, Hendrika H. van Hell, Gerry Jager, René S. Kahn, Nick F. Ramsey

**Affiliations:** 1 Rudolf Magnus Institute of Neuroscience, Department of Neurology and Neurosurgery, University Medical Center Utrecht, Utrecht, The Netherlands; 2 Institute of Psychiatry, Department of Psychosis Studies, King’s College London, London, United Kingdom; 3 Division of Human Nutrition, Wageningen University, Wageningen, The Netherlands; 4 Rudolf Magnus Institute of Neuroscience, Department of Psychiatry, University Medical Center Utrecht, Utrecht, The Netherlands; Bellvitge Biomedical Research Institute-IDIBELL, Spain

## Abstract

Evidence is increasing for involvement of the endocannabinoid system in cognitive functions including attention and executive function, as well as in psychiatric disorders characterized by cognitive deficits, such as schizophrenia. Executive function appears to be associated with both modulation of active networks and inhibition of activity in the default mode network. In the present study, we examined the role of the endocannabinoid system in executive function, focusing on both the associated brain network and the default mode network. A pharmacological functional magnetic resonance imaging (fMRI) study was conducted with a placebo-controlled, cross-over design, investigating effects of the endocannabinoid agonist Δ9-tetrahydrocannabinol (THC) on executive function in 20 healthy volunteers, using a continuous performance task with identical pairs. Task performance was impaired after THC administration, reflected in both an increase in false alarms and a reduction in detected targets. This was associated with reduced deactivation in a set of brain regions linked to the default mode network, including posterior cingulate cortex and angular gyrus. Less deactivation was significantly correlated with lower performance after THC. Regions that were activated by the continuous performance task, notably bilateral prefrontal and parietal cortex, did not show effects of THC. These findings suggest an important role for the endocannabinoid system in both default mode modulation and executive function. This may be relevant for psychiatric disorders associated with executive function deficits, such as schizophrenia and ADHD.

## Introduction

The endocannabinoid (eCB) system is a retrograde messenger system that regulates both excitatory and inhibitory neurotransmission, and consists of cannabinoid receptors and accompanying endogenous ligands [1]. Recently, the eCB system has emerged as a potential candidate for pharmacological targeting of psychiatric syndromes including addiction [2] and schizophrenia [3]. Importantly, the eCB system has been associated with executive functions which are also affected in various psychiatric disorders. Modulation of the eCB system by administration of cannabis or Δ9-tetrahydrocannabinol (THC), the main psychoactive component in cannabis and partial agonist of the cannabinoid CB1 receptor, impairs performance on various executive function paradigms that target high-level cognitive functions essential for goal-directed behavior [4]–[8].

Goal-oriented behavior has recently been associated with reduced neural activity in the default mode network (DMN), which mainly consists of the posterior cingulate cortex, medial prefrontal cortex, and bilateral inferior parietal lobules (including the angular gyrus) [9]–[11]. Failure to reduce DMN activity impairs performance on various cognitive tasks [12]–[15]. Moreover, psychiatric patients such as patients with schizophrenia or attention-deficit hyperactivity disorder (ADHD), who exhibit a strong decline in cognitive function, display an inability to deactivate the DMN during performance of executive function paradigms [11], [16]–[23]. Collectively, this suggests a role for the DMN in cognitive function deficits.

The aim of the present study was to elucidate the role of the eCB system in executive function, in terms of performance and brain activity in both the DMN and the task-related network. To this end, a pharmacological functional MRI (fMRI) study was performed with acute THC administration, using a placebo-controlled cross-over design and a continuous performance task paradigm with identical pairs (CPT-IP) in healthy subjects [24], [25]. This version of the CPT is characterized by a heavy reliance on executive function, as it requires fast and continuous updating of information while short-term memory load is relatively small [26]. Previous imaging studies using CPT-IP paradigms have shown activation of an executive system predominantly consisting of frontal and parietal regions [24], [25]. We compared performance on the CPT-IP task after placebo and after THC administration, and assessed the role of the DMN and the executive system in the effect of THC. On the basis of neuropsychological findings [4]–[7], it was expected that THC administration would reduce performance on the CPT-IP task. This was hypothesized to be associated with reduced deactivation of the DMN, as this has been shown to impair cognitive performance [12]–[15]. In addition, an increase in perceived erroneous responses may result in increased effort to maintain good performance levels, most likely reflected as elevated activity in the executive system [27], [28].

## Materials and Methods

This study is part of the Pharmacological Imaging of the Cannabinoid System (PhICS) project, the design and objectives of which are provided in a methodological paper [29].

### Ethics Statement

The study was approved by the Independent Ethics Committee of the University Medical Center Utrecht, the Netherlands, in accordance to the Declaration of Helsinki 2008. All volunteers gave written informed consent before entry into the study.

### Subjects

Twenty-three healthy male right-handed subjects were recruited through flyers, posters and internet advertisements. All subjects were incidental cannabis users, defined as having used cannabis at least four times but at most once a week in the year before inclusion in the study. All subjects were in good physical health as assessed by medical history and physical examination, and were screened for axis I psychiatric disorders using the Mini International Neuropsychiatric Interview for DSM-IV clinical disorders. Subjects were asked to refrain from cannabis for at least two weeks before the first study day until study completion. Illicit drug use other than cannabis was not within six months prior to inclusion. Compliance was tested by means of a urine sample at the beginning of each test day. Subjects needed to abstain from alcohol for 48 hours before each study day, and caffeine intake and smoking were not allowed from the moment of arrival until the end of a study day. For further details on inclusion and exclusion criteria we refer to Van Hell et al. [29].

Results are reported on twenty out of the twenty-three included subjects. One subject did not complete the study procedure due to high blood pressure levels. Two other subjects were excluded because of an absence of detectable THC plasma levels and technical malfunction during scanning, respectively. Subject characteristics are summarized in Table 1.

**Table 1:** Subject characteristics (n = 20).

Characteristic	Mean ± SD	Range
Age (years)	22.9±4.9	18–40
IQ	105.6±5.6	97–114
Height (cm)	185.9±7.9	175–201
Weight (kg)	77.0±11.3	60–110
BMI (kg/m2)	22.2±2.1	18.5–27.2
Cannabis use last year (Occasions)	22.5±15.2	4–52
Cannabis use lifetime (Occasions)	337±448	32–1415
Age of cannabis use onset	15.7±1.7	13–21
Years of cannabis use	7.3±5.1	1–25
Tobacco smoking (Cigarettes/week)	57.6±60.8	0–140
Alcohol consumption (Units/week)	12.5±7.8	2–30
Coffee consumption (Units/week)	17.4±12.4	0–40
Illicit drug use (Occasions lifetime)	2.0±4.0	0–17

Use of tobacco, alcohol and coffee was given for the year before inclusion in the study. Subjects refrained from cannabis for at least two weeks before the first study day until study completion and from alcohol for 48 hours before each study day. Caffeine intake and smoking were not allowed from the moment of arrival until the end of a study day. Illicit drug use other than cannabis was at least more than six months before the first study day. All subjects showed negative urine screening at both study days.

### Design and Procedure

Using a double-blind, randomised design, subjects underwent two scanning sessions: one with placebo administration and one with THC, balanced over subjects. As not all subjects could be included, eight of the twenty subjects received placebo first. Study days were scheduled two weeks apart to allow for complete clearance of drugs. On study days, subjects performed three cognitive paradigms, during which fMRI scans were obtained. One of these paradigms was the CPT-IP. Paradigm sequence was balanced over subjects, but remained unchanged within subjects across sessions. Results of other assessments are reported elsewhere [29]–[33]. Although there is some overlap in subjects participating in our current and previous studies, none of the published studies have identical experimental groups.

Subjects received subsequent doses of THC or placebo with 30 minutes intervals. Drugs were administered 7 minutes before the start of each fMRI task using a Volcano ® vaporizer (Storz-Bickel GmbH, Tuttlingen, Germany) [34], [35]. The first THC dose was 6 mg, followed by three doses of 1 mg each to maintain stable levels of CNS effects. See Van Hell et al. [29] for detailed study procedures.

### Drug Levels and Behavioral Measurements

Venous blood samples were collected 5 and 27 minutes after administration to determine plasma concentrations of THC and its two most important metabolites, 11-hydroxy-THC and 11-nor-9-carboxy-THC, and were processed according to Zuurman et al. [35].

Subjective effects were determined with two sets of visual analogue scales (VAS) [36], [37]. The first rating scale consisted of 16 VAS from which three factors were calculated, corresponding to alertness, contentedness, and calmness [36]. From a second set of 13 VAS [37], composite scores of ‘external perception’ and ‘internal perception’ were calculated, whereas ‘feeling high’ was analyzed individually, as validated by Zuurman and colleagues [31]. Computerized versions of both rating scales were performed consecutively at baseline and before and after task performance. VAS data were corrected for baseline values, and each set of VAS was analyzed with a multivariate approach to repeated measures ANOVA with factors drug (2 levels: placebo and THC), time (2 levels: before and after task performance) and scale (3 levels for each set of VAS). Post hoc repeated measures ANOVA was performed to further investigate effects on individual VAS items.

Heart rate was measured regularly at fixed intervals before scanning, and monitored continuously during scanning. Mean heart rate during scanning was calculated by dividing the total number of heart beat trigger signals by the duration of the CPT-IP task [38]. Mean heart rate during scanning was corrected for mean baseline values, and placebo and THC sessions were statistically compared with a paired t test.

### Task Paradigm

**Figure 1:**
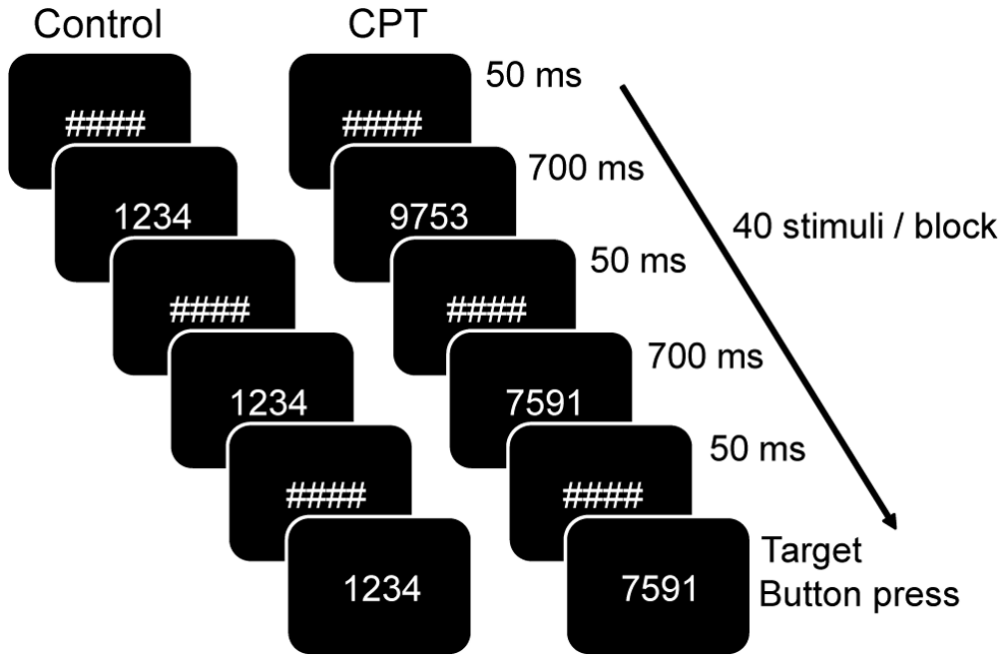
Schematic outline of the task used to assess executive function. The task consists of a control (CT, left) and an experimental condition (CPT-IP, right), during which four-digit numbers are presented in sequence. In the experimental condition, subjects have to press a button as quickly as possible when two consecutive numbers are identical. No response is required for the control condition. See for detailed information the Materials and methods section.

Executive function was assessed with a CPT with identical pairs (CPT-IP) consisting of two different task conditions (Figure 1) [24], [25]. In the experimental condition (CPT-IP), participants were presented with a series of four-digit numbers, and were instructed to press a button as quickly as possible when two consecutive numbers were identical. In a control task (CT), subjects were always presented with the same stimulus (‘1234’), and were instructed to watch the stimuli, but not to respond. This task was designed to control for the simple visual components of watching flashing numbers.

The CPT-IP and CT tasks were given in alternating blocks of 30 s each. Six blocks of each task were presented, together with six rest blocks. The order of blocks was counterbalanced within sessions. A total of 40 numbers per block was presented. Every number appeared for 700 ms, followed by a fixation cross of 50 ms. For the CPT-IP task only, the number of targets per block varied from seven to nine, with an average of eight targets per block. In addition, each CPT-IP block contained eight distracters, defined as numbers consisting of similar digits as the preceding number, but presented in another order. Total task duration was 11 minutes. Numbers differed for both study days for all subjects.

Outcome measures for the CPT-IP task included reaction time for hits (RT), the mean percentage of correctly identified targets (% hits), and the mean percentage of incorrectly identified targets (% false alarms). Group differences in RT and performance accuracy between placebo and THC were analyzed with paired t tests.

### Image Acquisition

Image acquisition was performed on a Philips Achieva 3.0 Tesla scanner (Philips Medical Systems, Best, the Netherlands). Functional images were obtained using a 3D PRESTO-SENSE pulse sequence [39] (parameters: scan time 0.6075 s; TR 22.5 ms (in contrast to EPI, for PRESTO the TR is much shorter than the time to scan one volume, see [39]; TE 33.2 ms; flip angle = 10°; FOV 224×256×160; matrix 56×64×40; voxel size 4 mm isotropic; 40 slices (sagittal orientation); 1105 volumes). A high-contrast volume with a flip angle 27° was scanned for registration purposes. A T1-weighted structural image was obtained for anatomical registration (parameters: TR 9.5 ms; TE 4.7 ms; flip angle = 8°; FOV 220.8×240×159.6; matrix 368×400×266; voxel size 0.6 mm isotropic, 266 slices (sagittal orientation)).

### Functional MRI Analysis

Functional MRI data were preprocessed and analyzed using SPM5 (Wellcome Trust Centre for Neuroimaging, London, UK). Preprocessing included realignment of functional images, co-registration with the anatomical volume using the flip angle of 27° volume, spatial normalization into standard MNI space, and smoothing (FWHM = 8 mm), as described previously [30], [31], [33]. There were no significant differences between sessions in scan quality in terms of the average standard deviation of time series.

First level single subject analysis included a general linear model regression analysis using a factor matrix with factors for the CPT-IP and CT condition, as well as the instructions that were presented during the task and factors to correct for slow drifts in the signal up to 0.004 Hz. Group activity maps were created for both the placebo and THC session for the CPT-IP minus CT contrast.

We chose to perform ROI analyses, because we expected the default mode and executive system to act as connected networks. In addition, this analysis (unlike voxel-wise whole brain analysis) allows for both calculation and presentation of effect sizes and follow-up analysis, and has sufficient power for smaller samples [40]–[42]. We preselected ‘task’ voxels that showed significant signal changes associated with the experimental paradigm (thresholded at |t| >4.6, p<0.0001). To prevent session bias in voxel selection, voxels were included if they exceeded threshold in either the placebo or THC session. Regions of interest (ROIs) were identified by clustering groups of at least ten neighboring active voxels (640 mm^3^). We chose a lenient threshold for voxels selection to ensure that we included most regions showing signal changes related to the task. Notably, the threshold for ROI identification has no direct relationship to the tested experimental hypotheses [40].

ROIs were divided in two groups: ROIs showing task-related increases are referred to as task-induced activation (‘TIA’) ROIs. ROIs based on voxels showing signal decrease are referred to as task-induced deactivation (‘TID’) ROIs. Mean signal change for each ROI, each subject, and each session (placebo and THC) was based on regression coefficients (b values) for the CPT-IP condition averaged over voxels in each ROI, extracted using the Marsbar SPM tool [43].

It is at this stage that statistical hypothesis testing was conducted, using SPSS 17. To measure THC effects on brain activity in TIA ROIs, a multivariate approach to repeated measures ANOVA was used with drug (2 levels: placebo and THC) and ROI (15 levels: all TIA regions included) as within-subject factors. To measure THC effects on brain activity in TID ROIs, a multivariate approach to repeated measures ANOVA was used with drug (2 levels) and ROI (4 levels: TID regions) as within-subject factors (see the results section for details about ROIs).

To directly compare effects of THC in TIA and TID networks, mean regression coefficients for the CPT-IP condition were averaged over all included voxels for either network, for both the placebo and THC condition. To measure THC effects on network activity, a multivariate approach to repeated measures ANOVA was used with drug (2 levels: placebo and THC) and network (2 levels: TIA and TID network) as within-subject factors.

### Correlation Analyses

For further understanding of the acute effects of THC on executive function, correlation analyses were performed between task performance (percentage of correct responses, which reflects both hits and false alarms), brain activity (TID and TIA network), peak plasma concentrations of THC and its two main metabolites, and subjective effects after THC administration (Pearson’s r). Follow up analyses are presented as a further descriptive exploration of the main hypothesis test, and are, as such, not corrected for multiple comparisons.

## Results

### Drug Levels and Behavioral Measurements

Plasma concentrations of THC and its main metabolites were 78.4±27.0 ng/ml (THC), 3.9±4.6 ng/ml (11-nor-9-carboxy-THC) and 2.5±2.0 ng/ml (11-hydroxy-THC), 5 min after inhalation of 6 mg THC.

Overall repeated measures ANOVA analysis of the VAS scales ‘feeling high’, ‘internal perception’ and ‘external perception’ [37] revealed a significant difference in the drug by time interaction between VAS scales (drug * time * VAS scale interaction, F(2,18) = 7.90, p = 0.003), a significant difference in the effect of THC both between VAS scales (drug * VAS scale interaction, F(2,18) = 11.44, p = 0.001) and between time points (drug * time interaction, F(1,19) = 8.37, p = 0.009), a significant increase in VAS score with THC administration (drug effect, F(1,19) = 15.06, p = 0.001), and a higher VAS score after task performance (time effect, F(1,19) = 16.01, p = 0.001). Post hoc analysis of the VAS scale ‘feeling high’ showed a significant increase in VAS score with THC administration (drug effect, F(1,19) = 19.10, p<0.001) and a higher VAS score after task performance (time effect, F(1,19) = 7.39, p = 0.014), without differences in the effects of THC between time points (drug * time interaction, F(1,19 = 3.64, p = 0.072). Analysis of ‘external perception’ (reflecting misperception of external stimuli or changes in the awareness of the environment) showed a significant increase in VAS score with THC administration (drug effect, F(1,19) = 11.03, p = 0.004) and a higher VAS score after task performance (time effect, F(1,19) = 8.11, p = 0.010), with a significant difference in the effect of THC between time points (drug * time interaction, F(1,19 = 13.09, p = 0.002). Post hoc analysis of the VAS scale ‘internal perception’ (reflecting inner feelings that do not correspond with reality) did not show any significant effects (see Table 2).

**Table 2:** Subjective effects of Δ9-tetrahydrocannabinol (THC) (n = 20).

	Repeated measures ANOVA effects (F(1,19))				
VAS Assessment	Drug	Time	Drug* Time	Mean Placebo score (± SD)	Mean THC score (± SD)
Feeling High	**19.10, p<0.001** *	**7.39, p = 0.014** *	3.64, p = 0.072	2.63±6.41	27.00±25.99
Internal Perception	3.42, p = 0.080	2.36, p = 0.142	2.28, p = 0.148	0.15±0.63	3.15±7.06
External Perception	**11.03, p = 0.004** *	**8.11, p = 0.010** *	**13.09, p = 0.002** *	0.98±2.24	9.15±10.29
Alertness	**9.24, p = 0.007** *	**15.58, p = 0.001** *	0.10, p = 0.756	−7.44±7.68	−17.03±12.72
Contentedness	**10.03, p = 0.005** *	1.13, p = 0.300	0.49, p = 0.491	−3.60±8.05	−11.68±9.73
Calmness	**10.10, p = 0.005** *	0.071, p = 0.793	3.17, p = 0.091	4.94±12.82	−9.63±18.20

Statistical analysis was performed with baseline corrected values using a multivariate approach to repeated measures ANOVA with drug and time as factors.

* Significant difference between placebo and THC (p<0.05). VAS, Visual Analogue Scale.

Overall repeated measures ANOVA analysis of the VAS scales ‘alertness’, ‘contentedness’, and ‘calmness’ [36] revealed a significant increase in VAS score with THC administration (drug effect, F(1,19) = 12.24; p = 0.002), without any significant differences in VAS scores between time points or in the effects of THC between VAS scales or time points (time effect, F(1,19) = 1.66, p = 0.213; drug * VAS scale interaction, F(2,18) = 2.24, p = 0.135; drug * time interaction, F(1,19) = 1.67, p = 0.212; drug * time * VAS scale interaction, F(2,18) = 1.16; p = 0.337). Post hoc analysis of individual VAS scales showed a significant decrease in VAS score with THC administration for ‘alertness’ (drug effect, F(1,19) = 9.24, p = 0.007), ‘contentedness’ (drug effect, F(1,19) = 10.03, p = 0.005), and ‘calmness’ (drug effect, F(1,19) = 10.10, p = 0.005). The VAS score on ‘alertness’ was significantly lower after task performance (time effect, F(1,19) = 15.58, p = 0.001). Results of VAS post hoc analyses are summarized in Table 2.

Heart rate increased significantly after THC compared with placebo (22.2±14.5 and −1.5±7.8 bpm increase compared to baseline (± SD), respectively; t(17) = −6.85, p<0.001). For a more detailed description of drug levels and behavioral measurements following THC see Van Hell et al. [29].

### Task Performance

**Figure 2:**
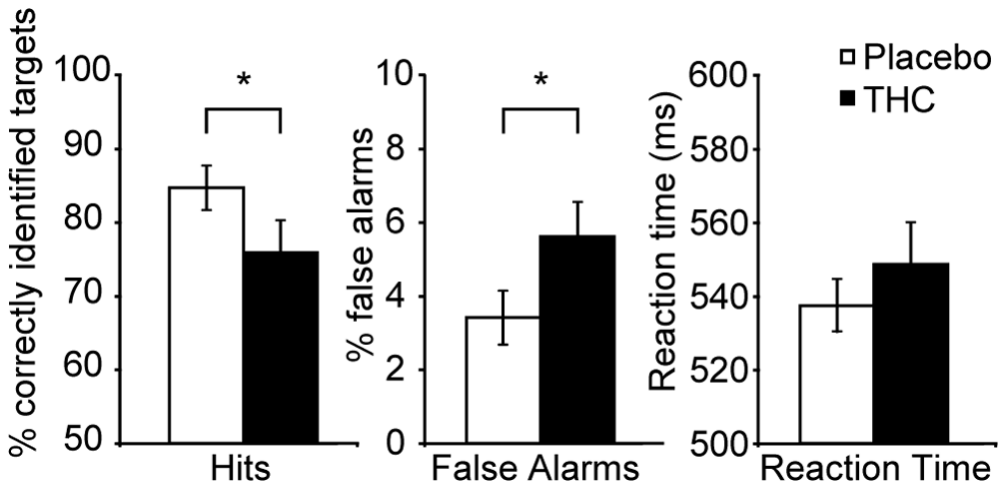
Task performance. The figure shows (left) the mean percentage of correctly identified targets, (middle) the mean percentage of false alarms, and (right) reaction times of correct responses after placebo and THC administration (n = 20; mean ± SEM). * Significant difference between THC and placebo (p<0.05).

THC administration significantly decreased the percentage of correctly identified targets (from 83.7±2.9% to 74.7±4.3%, t(19) = 2.66, p = 0.016) and enhanced the percentage of false alarms (from 3.5±0.7% to 5.7±0.9%, t(19) = −3.76, p = 0.001). Reaction times on the CPT-IP did not differ between placebo and THC sessions (538.5±7.1 and 552.0±11.0 ms, respectively; t(19) = −1.07, p = 0.296) (all ± SEM, see Figure 2).

### Selection of Regions of Interest

**Figure 3:**
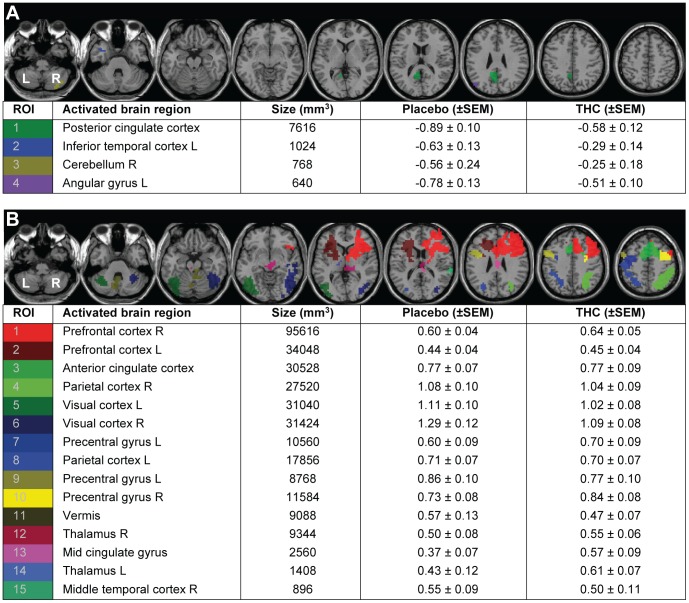
Effects of THC administration on activity in regions of interest (ROIs). The figure shows **A**, task-induced deactivation (TID), and **B**, task-induced activation (TIA). ROIs are defined in CPT-IP minus CT group activity maps, pooled over placebo and THC (n = 20; t>|4.6|, p<0.0001 uncorrected, clusters ≥10 voxels). L, left; R, right.

Task activity was measured in a set of regions showing task-induced deactivation (TID) and a set of regions showing task-induced activation (TIA). TID showed a network of four regions, comprising posterior cingulate cortex, left inferior temporal gyrus, right cerebellum and left angular gyrus (Figure 3A). TIA yielded a network of 15 brain regions, comprising bilateral prefrontal cortex, parietal cortex, precentral gyrus, visual cortex, and thalamus, as well as anterior cingulate cortex, mid cingulate gyrus, vermis, and right middle temporal cortex (Figure 3B).

### Effects of THC on Task-induced Deactivation

**Figure 4:**
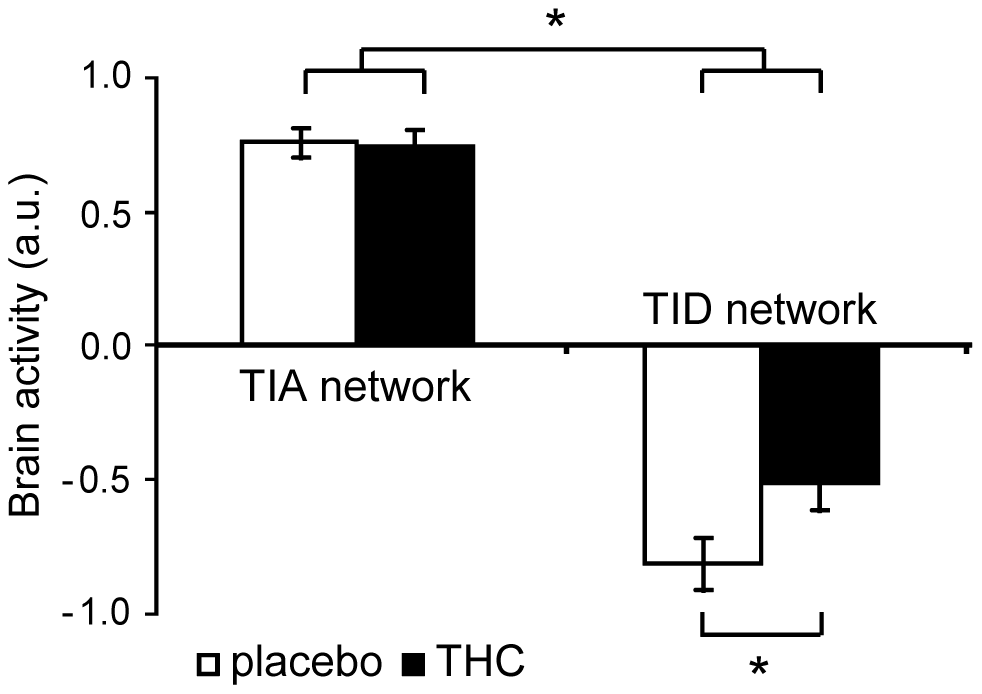
Brain activity in the TIA (left) and TID network (right, all voxels combined). The figure shows activity after administration of placebo (white) and THC (black) (n = 20; mean ± SEM). A significant interaction effect between drug and network indicates that THC had a different effect on activity in the TID than in the TIA network. * Significant effect (p<0.05). TIA, task-induced activation; TID, task-induced deactivation; a.u., arbitrary units.

Activity in TID regions was significantly increased after THC administration (F(1,19) = 13.20; p = 0.002) (Figure 4, right). There was no significant drug * ROI interaction in TID ROIs (F(1,19) = 0.06, p = 0.98) (Figure 3A, Figure S1A, Figure S2).

### Effects of THC on Task-induced Activation

Brain activity in TIA regions was not affected by THC administration (F(1,19) = 0.02; p = 0.90), indicating that THC did not induce a change in the pattern of TIA activity during CPT-IP (Figure 4, left). There was no significant difference in the effect of THC between TIA ROIs (drug * ROI interaction, F(1,19) = 0.72, p = 0.71) (Figure 3B, Figure S1B, Figure S2).

### Task-induced Activation vs Task-induced Deactivation

**Figure 5:**
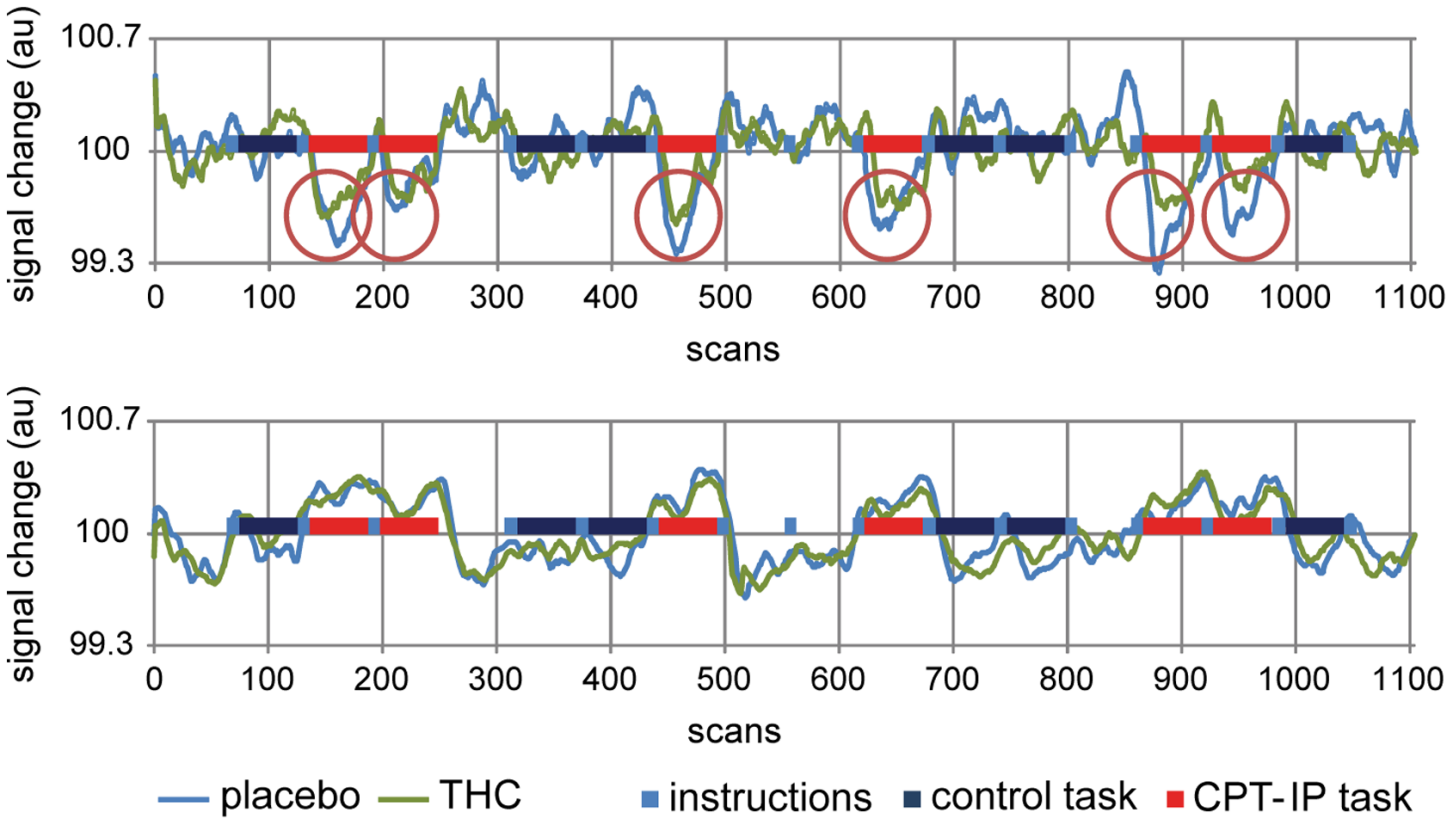
Activity over time in the TID (upper graph) and TIA network (lower graph) during CPT-IP performance. The figure shows activity after placebo (blue) and THC (green) administration (n = 20; mean). The upper graph demonstrates the consistently smaller deactivation in the TID network after THC administration, while the lower graph shows that activity in TIA ROIs is virtually unchanged after THC. TIA, task-induced activation; TID, task-induced deactivation; au, arbitrary units.

A direct comparison of THC effects on the TIA and TID networks, using the average activity in each network, revealed a significant interaction effect between drug and network (F(1,19) = 6.97; p = 0.02), reflecting that the TID network was more sensitive to the effects of THC than the TIA network (Figure 4, Figure 5).

### Correlations

**Figure 6:**
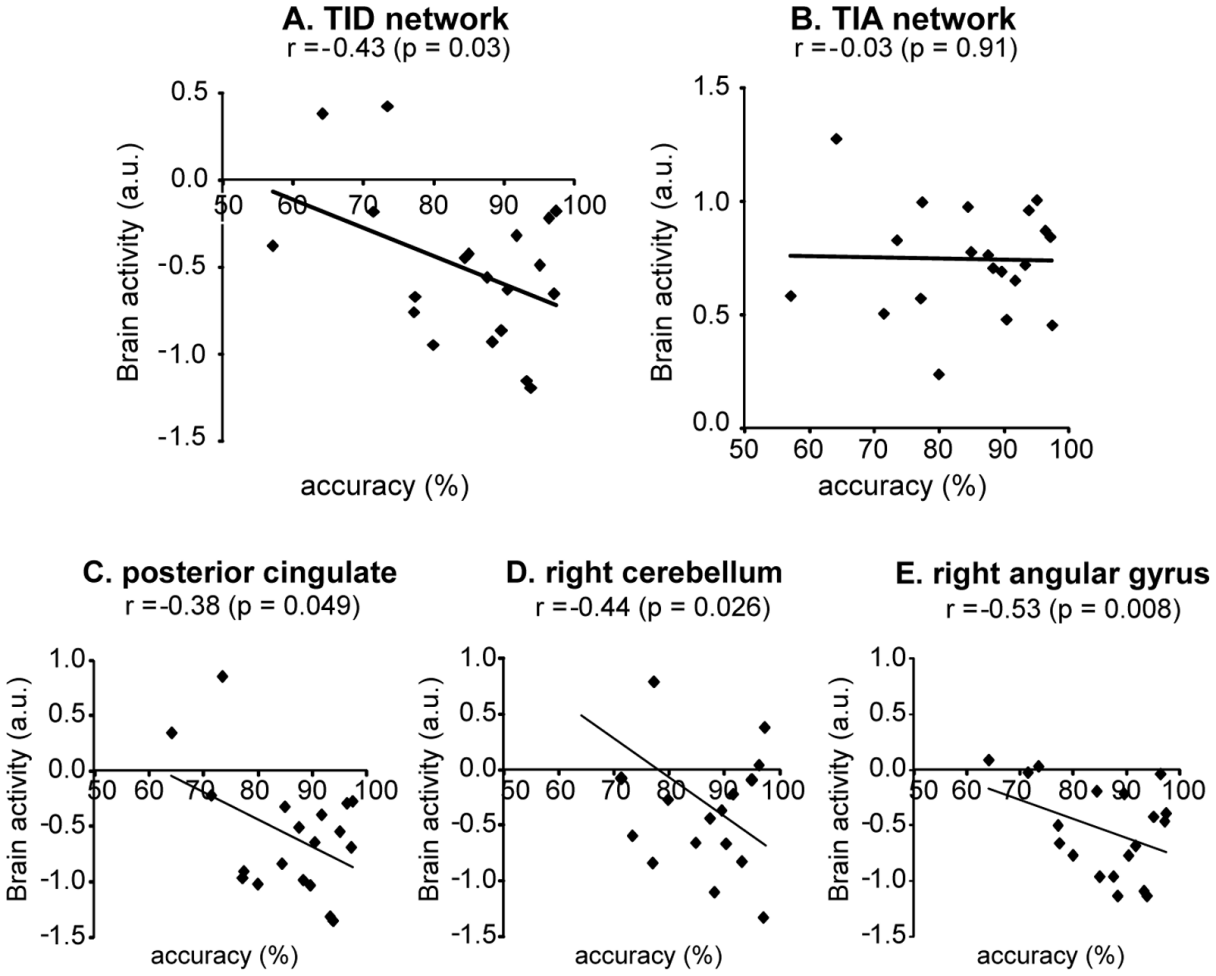
Correlations between performance (percentage correct responses) and brain activity. The figure shows correlations in **A**, TID network, **B**, TIA network, **C**, posterior cingulate cortex, **D**, right cerebellum, and **E**, right angular gyrus. TIA, task-induced activation; TID, task-induced deactivation; a.u., arbitrary units.

Task performance (percentage of correct responses, see Figure S3) showed a significant negative correlation with activity in the TID network after THC (r = −0.43, p = 0.03) (Figure 6). Follow up analysis in the four TID ROIs indicated a significant negative correlation in posterior cingulate cortex (r = −0.38, p = 0.049), right cerebellum (r = −0.44, p = 0.026) and left angular gyrus (r = −0.53, p = 0.008). No significant correlation was found between performance and TIA activity (r = −0.03; p = 0.91) (Figure 6).

Peak THC plasma concentration showed a significant negative correlation with activity in the TID network after THC (r = −0.55, p = 0.007). Follow up ROI analysis in the four TID ROIs indicated a significant negative correlation in posterior cingulate cortex (r = −0.43, p = 0.033), right cerebellum (r = −0.42, p = 0.038) and left angular gyrus (r = −0.58, p = 0.004). No significant correlation was found between peak THC plasma concentration and TIA activity (r = −0.32; p = 0.186). Levels of 11-OH-THC and 11-nor-9-carboxy-THC did not show significant correlations with either VAS subjective ratings, task performance or network activity. Subjective effects did not show significant correlations with other measures of interest.

## Discussion

The role of the eCB system in executive function was studied in an fMRI study with a THC challenge, focusing on processing of continuously updated information and the role of DMN. After THC administration, subjects showed impaired task performance, reflected in both an increase in false alarms and a reduction in detected targets. Brain regions that were deactivated during the task showed less deactivation after THC than after placebo. In addition, after THC, task performance was negatively correlated with activity in the deactivated regions. In contrast, regions that were positively activated by the task did not show THC-induced changes in activity. Together, these results indicate that the DMN plays a role in the effects of THC on executive function. Effects of THC on DMN activity were predominantly found in the posterior cingulate cortex and angular gyrus, both considered pivotal DMN nodes [9]–[11].

A possible role of DMN in executive function is addressed by the default mode interference hypothesis which states that functions performed by the DMN interfere with successful goal-oriented performance [44]. In the context of a normally functioning brain, the DMN component is attenuated during goal-directed action, and the level of attenuation is independent of task content. Neuroimaging studies support this notion, as several studies have linked reduced DMN activity with successful task execution [12]–[15]. In addition, the level of reduction in DMN activity appears to reflect the relative resources that need to be allocated to task execution [45], [46]. How exactly interference occurs is largely unknown, but a possibility is that DMN functions use similar resources as those needed for goal-oriented behavior. Possible functions of the DMN include conscious processes that occur in the absence of goal-oriented behavior, such as self-referential mental processing [47], mind-wandering [48], and mental explorations and simulations [10].

To our best knowledge, this is the first study that shows effects of THC administration on task-induced deactivations. In line with our finding of a lower degree of deactivation in the posterior cingulate cortex after THC administration, a recent resting state fMRI study showed that THC decreased functional connectivity between the posterior cingulate cortex and a network of brain regions collectively referred to as the left dorsal visual stream, which is thought to be involved in attentional processes [49].

In the present study, subjective ratings of ‘feeling high’ and ‘external perception’ were increased, whereas those of ‘alertness’, ‘contentedness’ and ‘calmness’ were reduced after THC administration. Similar behavioral effects of THC on feeling high, external perception and alertness have previously been described [35]. Decreases in contentedness and calmness may be related to THC administration in an MRI environment, as they were not shown in a previous study using Positron Emission Tomography (PET) [34].

THC-induced effects on DMN activity suggest eCB involvement in regulation of default mode activity. A potential neurobiological explanation may be found in the ‘on-demand’ modulating role of the eCB system in neurotransmitter release. This eCB-mediated regulation of synaptic transmission is a widespread phenomenon in the brain, and is thought to play an important role in higher cognitive functions [1]. THC administration may disrupt this function of the eCB system [50]. Recently, it has been shown that negative BOLD responses are tightly coupled to reductions in neuronal activity [51], most likely mediated by increased GABA transmission in the DMN [52]. Importantly, increasing cognitive load was associated with more DMN deactivation and higher GABA concentrations [52]. This suggests that THC administration may affect DMN activity through disruption of eCB-mediated GABA neurotransmission.

Our results may have implications for understanding impairments in executive function related to psychiatric and neurological disorders. For example, several studies have shown that schizophrenia patients exhibit an inability to deactivate the DMN during various cognitive tasks [16], [17]. Impairment in capacity to reduce DMN activity has also been identified in other patient groups [20]–[23], such as youth with ADHD [18], [19] and patients with Alzheimer’s disease [53]. Our results suggest that the eCB system may be a factor in the abnormal DMN activity associated with aforementioned disorders, and, as such, could be involved in cognitive deficits in these disorders.

The current study demonstrated an extensive set of regions that was positively activated by the task. Previous imaging studies using executive function paradigms have shown activation of a similar network [24], [25], also referred to as the Central Executive System (CES) [54], which has been associated with several functions necessary for successful executive function, such as the detection and selection of sensory stimuli [55], the subsequent linking of stimuli to appropriate motor responses [56], and the ability to detect erroneous responses [27], [28]. Consistent with this latter CES function, THC-induced impairment of task performance as shown in the present study is expected to result in increased effort to maintain good performance levels, most likely reflected as elevated CES activity. However, THC did not affect CES activity during performance of the task. This suggests that under influence of THC, subjects may have been unaware of their impaired performance, thereby dismissing the need for elevated CES activity. Consistently, chronic cannabis users, who showed good task performance, demonstrated diminished capacity for error monitoring which was associated with reduced CES activity [57].

Previous studies have reported reduced activity in the CES in psychiatric disorders such as ADHD [58], [59] and schizophrenia [60], [61], an effect that is likely related to impaired task performance [61]. One explanation for the apparent discrepancy with the current findings could be that performance deficits as shown in our study after THC are moderate compared to those of psychiatric patients. For example, decreased CES activity in schizophrenia patients in the study of Salgado-Pineda et al. [60] was associated with a 33% reduction in the mean percentage of correctly identified targets. This view is further supported by studies in which CES activity of schizophrenia patients was not reduced during adequate performance of moderately difficult central executive tasks [62], [63].

DMN-related brain activity has been shown to be affected by human genetic variation, such as functional polymorphisms in the catechol-O-methyltransferase (COMT) gene. Higher COMT activity, resulting in reduced prefrontal dopamine neurotransmission, has been associated with significantly greater deactivation of the posterior cingulate cortex of healthy volunteers [64] and reduced deactivation in the medial prefrontal cortex of both healthy subjects and schizophrenia patients [65] during performance of executive function tasks. A recent multimodal neuroimaging study demonstrated that genetic variation in the dopamine D2 receptor (DRD2) gene modulates connectivity strength within the DMN during a working memory task, which was associated with striatal dopamine transporter availability as measured with Single Photon Emission Computed Tomography (SPECT) [66]. Interestingly, individuals with increased COMT activity appear to have stronger responses to THC administration in terms of acute psychotic effects and cognitive impairments [67]. Altogether, these findings suggest the possibility that the effect of THC on DMN activity as shown in the present study may depend on individual genetic profiles, particularly of genes involved in dopamine neurotransmission.

An increasing number of imaging studies use a pharmacological challenge to study effects on cognition. For instance, the norepinephrine/dopamine transporter inhibitor modafinil reduced DMN activity during a simple visuomotor task. The modafinil effect in the ventromedial prefrontal cortex was significantly correlated with reaction time [68]. Treatment with methylphenidate normalized DMN activity in off-methylphenidate ADHD patients who showed attenuated DMN activity during low incentive conditions [69]. In addition, nicotine administration decreased DMN activity at rest in non-smokers [70], improved cognitive withdrawal symptoms of abstinent smokers through modulation of functional connectivity within the DMN and of inverse coupling between default mode and central executive brain networks [71], and enhanced visuospatial attention by deactivating DMN nodes including posterior cingulate cortex and angular gyrus in minimally deprived smokers [15]. These studies provide converging evidence for an important role of DMN in cognitive performance.

This study has several limitations. First, our experimental paradigm focused on one aspect of executive function, namely processing of continuously updated information. Current results cannot be generalized over other aspects of executive function, such as dual task execution, inhibition, and selective attention. Second, although subjects were instructed to watch all stimuli that were presented in the control task of our experimental paradigm, this cannot be ensured as this task did not require a button press. As a result, not only regions involved in executive function, but also areas subserving motor and visual responses may be included in the network of regions that were positively activated by the task. It is unlikely, however, that this has affected our results as individual regions included in this network did not show significant effects of THC administration. Third, inclusion of incidental cannabis users, as opposed to non-users, may affect interpretation of results as previous cannabis use may influence the eCB system. However, this seems less plausible as neither brain activity nor behavioral effects were significantly correlated with reported cannabis use (data not shown). Fourth, inclusion of both tobacco users and non-users may affect interpretation of results as tobacco use may influence DMN activity [15]. However, this is unlikely as there was no significant difference in the effect of THC on DMN activity between tobacco users and non-users, with both groups showing a similar direction of the effect. In addition, tobacco users did not show a significant correlation between the number of cigarettes used per week and the effect of THC on DMN activity (see Results S1). Fifth, the performance of ROI analyses implies that we could have missed effects of THC administration in non-task-specific areas or THC-induced shifts in activity within ROIs. Finally, non-specific THC-induced changes on cerebral blood flow may have confounded our results [72]. However, the correlation between DMN activity and performance after THC administration indicates that effects are specifically related to task execution.

In conclusion, this study shows that THC administration results in less deactivation in the DMN during an executive function task, an effect that is correlated with task performance. These results suggest an important role for the eCB system in both DMN modulation and executive function. The association of the eCB system with DMN modulation may be relevant for psychiatric disorders associated with executive function deficits, such as schizophrenia and ADHD, as well as for neurological disorders such as Alzheimer’s disease.

## Supporting Information

Figure S1 **Effects of THC administration on activity in regions of interest (ROIs).** The figure shows brain activity in **A**, TID regions, and **B**, TIA regions, after administration of placebo (white) and THC (black) (n  =  20; mean ± SEM). Full ROI names are given in Figure 3. TIA, task-induced activation; TID, task-induced deactivation; a.u., arbitrary units.
(PDF)

Figure S2 **Activity patterns during performance of CPT-IP (baseline: rest).** The figure shows activity after administration of **A**, placebo, and **B**, THC (n  =  20; t > |4.6|, p < 0.0001 uncorrected, clusters ≥ 10 voxels).
(PDF)

Figure S3 **Task performance in percentage of correct responses after placebo and THC administration (n  =  20; mean ± SEM).** THC administration significantly decreased the percentage of correct responses (from 90.5 ± 1.7% to 85.0 ± 2.5%, t(19)  =  2.95, p  =  0.008).
(PDF)

Results S1 (PDF)
