# Supplementary material for: Default Mode Network in the Effects of Δ9-Tetrahydrocannabinol (THC) on Human Executive Function
Source: PLoS One. 2013 Jul 31;8(7):e70074. doi: 10.1371/journal.pone.0070074 (PMC3729458; doi:10.1371/journal.pone.0070074)
Supplement: Figure S1 — Effects of THC administration on activity in regions of interest (ROIs). The figure shows brain activity in A, TID regions, and B, TIA regions, after administration of placebo (white) and THC (black) (n = 20; mean ± SEM). Full ROI names are given in Figure 3. TIA, task-induced activation; TID, task-induced deactivation; a.u., arbitrary units. (PDF) [file pone.0070074.s001.pdf]

**Figure S1**

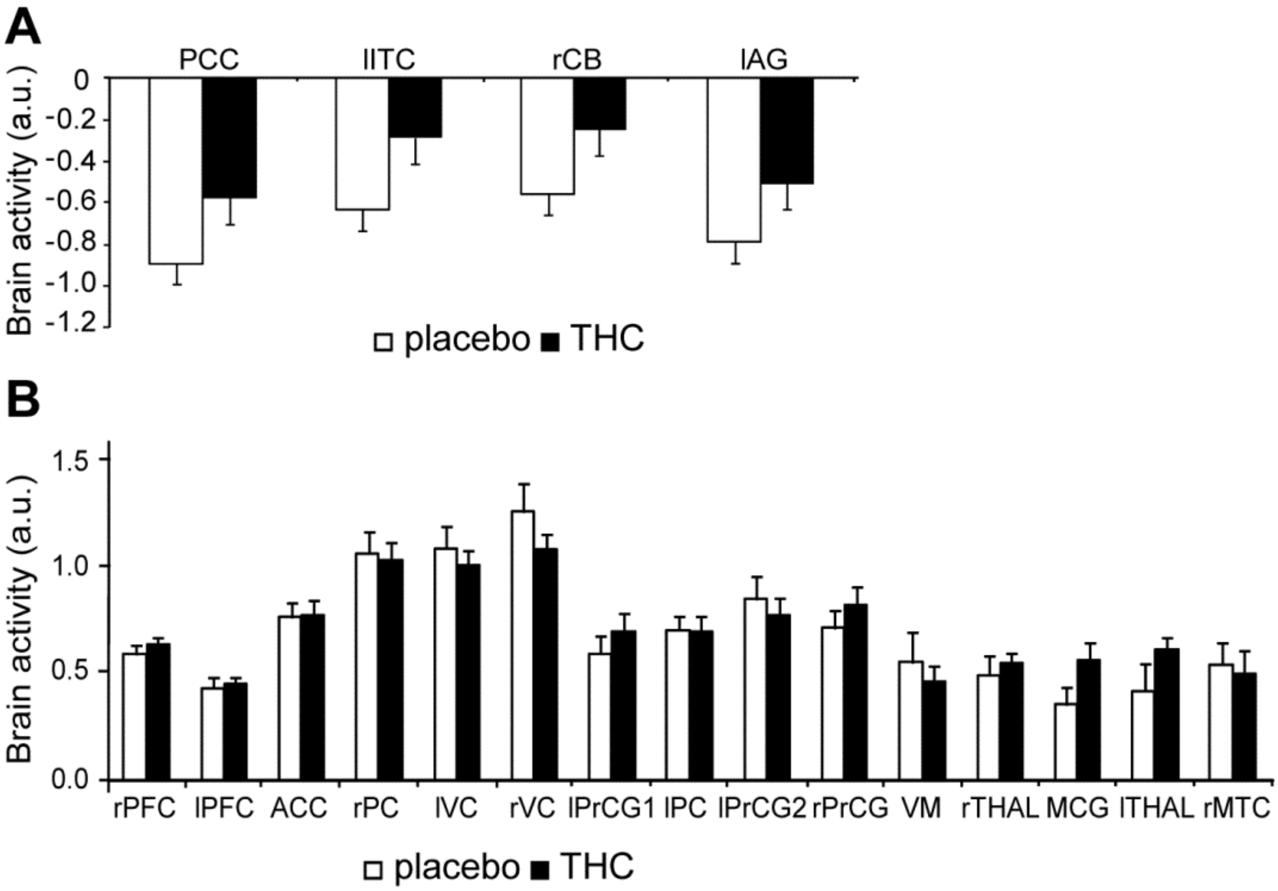


**Figure S1** Effects of THC administration on activity in regions of interest (ROIs). The figure shows brain activity in **A**, TID regions, and **B**, TIA regions, after administration of placebo (white) and THC (black) (n = 20; mean ± SEM). Full ROI names are given in Figure 3. TIA, task-induced activation; TID, task-induced deactivation; a.u., arbitrary units.
