# Supplementary material for: Default Mode Network in the Effects of Δ9-Tetrahydrocannabinol (THC) on Human Executive Function
Source: PLoS One. 2013 Jul 31;8(7):e70074. doi: 10.1371/journal.pone.0070074 (PMC3729458; doi:10.1371/journal.pone.0070074)
Supplement: Figure S2 — Activity patterns during performance of CPT-IP (baseline: rest). The figure shows activity after administration of A, placebo, and B, THC (n = 20; t > |4.6|, p < 0.0001 uncorrected, clusters ≥ 10 voxels). (PDF) [file pone.0070074.s002.pdf]

Figure S2

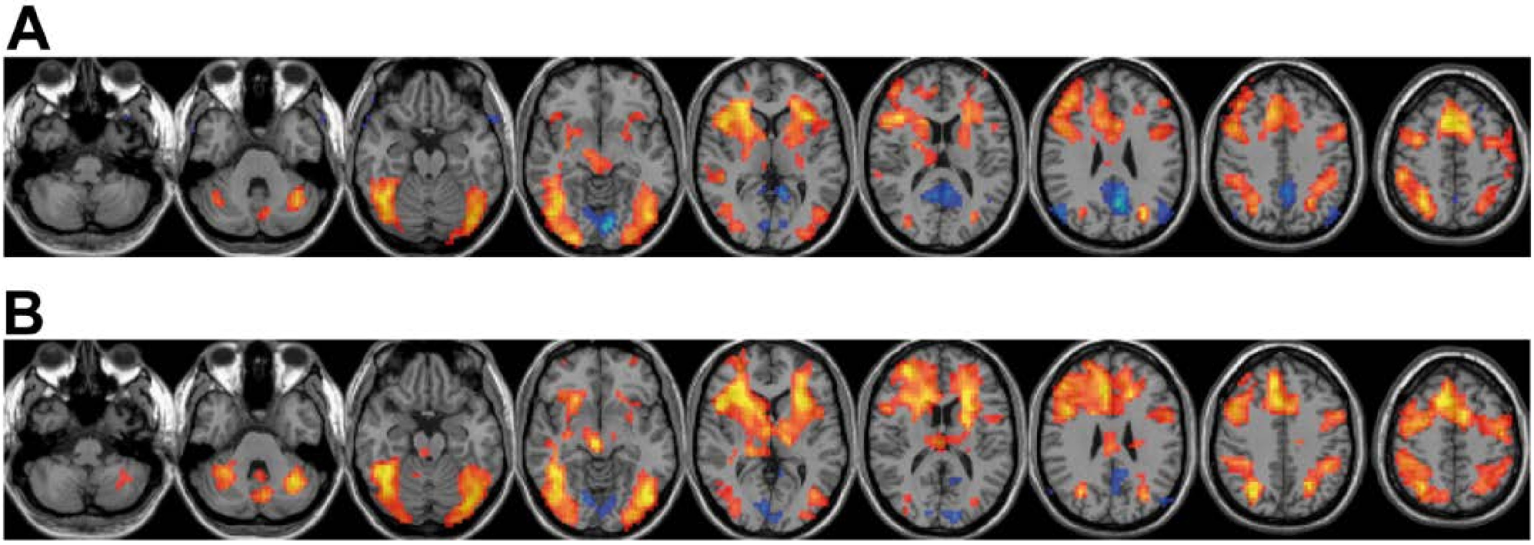

Figure S2 Activity patterns during performance of CPT-IP (baseline: rest). The figure shows activity after administration of **A**, placebo, and **B**, THC ($n = 20$; $t > |4.6|$, $p < 0.0001$ uncorrected, clusters ≥ 10 voxels).
