# Supplementary material for: Default Mode Network in the Effects of Δ9-Tetrahydrocannabinol (THC) on Human Executive Function
Source: PLoS One. 2013 Jul 31;8(7):e70074. doi: 10.1371/journal.pone.0070074 (PMC3729458; doi:10.1371/journal.pone.0070074)
Supplement: Figure S3 — Task performance in percentage of correct responses after placebo and THC administration (n = 20; mean ± SEM). THC administration significantly decreased the percentage of correct responses (from 90.5 ± 1.7% to 85.0 ± 2.5%, t(19) = 2.95, p = 0.008). (PDF) [file pone.0070074.s003.pdf]

Figure S3

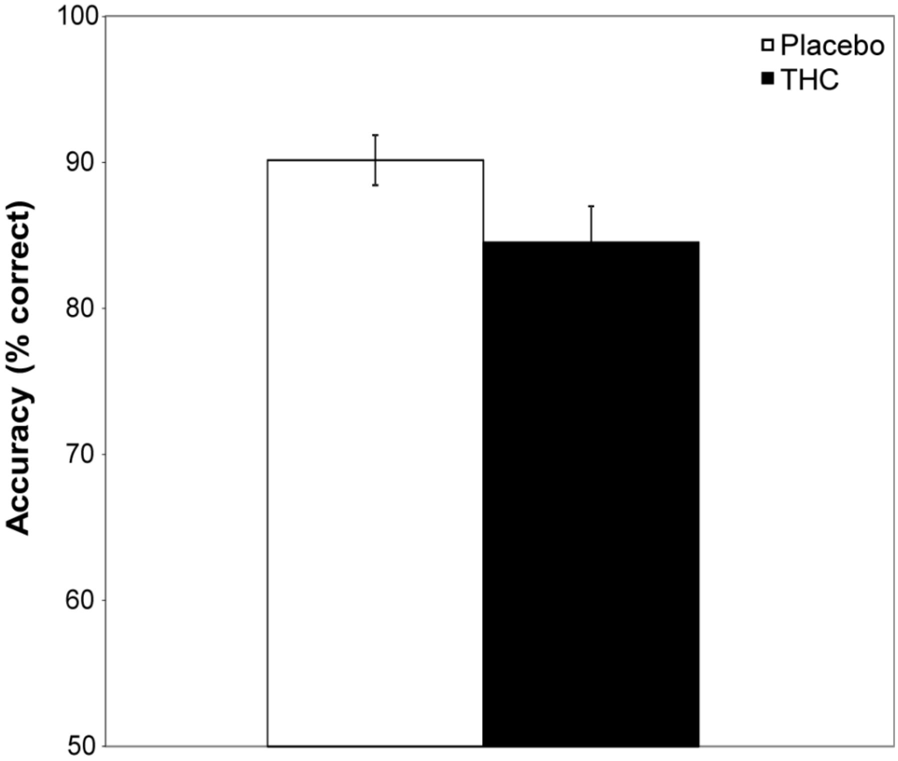

Figure S3 Task performance in percentage of correct responses after placebo and THC administration ($n = 20$; mean ± SEM). THC administration significantly decreased the percentage of correct responses (from 90.5 ± 1.7% to 85.0 ± 2.5%, $t(19) = 2.95$, $p = 0.008$).
