# Supplementary material for: Default Mode Network in the Effects of Δ9-Tetrahydrocannabinol (THC) on Human Executive Function
Source: PLoS One. 2013 Jul 31;8(7):e70074. doi: 10.1371/journal.pone.0070074 (PMC3729458; doi:10.1371/journal.pone.0070074)
Supplement: Results S1 — (PDF) [file pone.0070074.s004.pdf]

Results S1

To further explore the role of cigarette smoking in our results we compared the effect of THC on DMN activity between cigarette smokers (N=9, >70 cigarettes / week in the year before inclusion) and non-smokers (N=11). Performance of a repeated measures ANOVA with drug (2 levels: placebo and THC) and ROI (4 levels) as within-subject factors and group (2 levels: cigarette smokers and non-smokers) as between-subject factor did not show a significant drug * group interaction ($F(1,18) = 0.72$, $p = 0.406$). In addition, the direction of the effect of THC on DMN activity was similar in both groups (less DMN activity), and no correlation between number of cigarettes per week and the effect of THC on DMN activity (THC vs placebo) was shown in cigarette smokers (Pearson's $r = -0.006$, $p = 0.989$, two-sided).
